# Development of a Hybrid Piezo Natural Rubber Piezoelectricity and Piezoresistivity Sensor with Magnetic Clusters Made by Electric and Magnetic Field Assistance and Filling with Magnetic Compound Fluid

**DOI:** 10.3390/s17020346

**Published:** 2017-02-10

**Authors:** Kunio Shimada, Norihiko Saga

**Affiliations:** 1 Faculty of Symbiotic Systems Sciences, Fukushima University, 1 Kanayagawa, Fukushima 960-1296, Japan; 2 Department of Human System Interaction, Kansai Gakuin University, 2-1 Gakuen, Sanda, Hyogo 669-1337, Japan; saga@kwansei.ac.jp

**Keywords:** sensor, piezoelement, piezoelectricity, piezoresistivity, hybrid, electrolytic polymerization, magnetic field, magnetic cluster, natural rubber, magnetic compound fluid (MCF), magnetic fluid, isoprene, filler

## Abstract

Piezoelements used in robotics require large elasticity and extensibility to be installed in an artificial robot skin. However, the piezoelements used until recently are vulnerable to large forces because of the thin solid materials employed. To resolve this issue, we utilized a natural rubber and applied our proposed new method of aiding with magnetic and electric fields as well as filling with magnetic compound fluid (MCF) and doping. We have verified the piezoproperties of the resulting MCF rubber. The effect of the created magnetic clusters is featured in a new two types of multilayered structures of the piezoelement. By measuring the piezoelectricity response to pressure, the synergetic effects of the magnetic clusters, the doping and the electric polymerization on the piezoelectric effect were clarified. In addition, by examining the relation between the piezoelectricity and the piezoresistivity created in the MCF piezo element, we propose a hybrid piezoelement.

## 1. Introduction

Many types of sensors (force, temperature, pressure, etc.) are currently used in many different fields. Most sensors, including thermocouples and strain gauges, require a power supply in order for the electric signal of temperature or strain to be responsive to the voltage or electric current applied to the sensor. However, piezoelements have the distinguishing feature that they can sense an induced voltage without a power supply. Because in robot construction extra weight is very undesirable, power supplies should be avoided when possible, so in robotics, piezoelements are generally suitable for sensing force or pressure.

In addition, a piezoelement must be suitable for use in the artificial skin, which has large elasticity and extensibility, installed as an outer layer on robots. It is expected that in the future, robots will become a part of our lives as domestic help for chores, and in the nursery, hospital, etc. There is currently a need for a material that can serve as skin that is similar to human skin, because it is important that we feel affinity with robots. Therefore, when the piezoelement is made of a polymer [1,2,3,4], the polymer selected should be rubber, and not be vulnerable to impulsive or large forces. This is why it is necessity to use some form of rubber to create robot skins. Rubber is durable enough to withstand impulsive, large or repeated forces, while maintaining good elasticity and extensibility [5].

Many studies have sought to make rubber piezoelements. Silicon oil rubber has been the most commonly used material for piezoelements [6,7,8,9,10,11]. There have been a few investigations of other rubber materials: chloroprene [12], styrene-butadiene [13], acrylonitrile butadiene [14] and natural rubber [15]. In order to further improve the performance of a rubber sensor, it is often effective to apply a filler in the rubber. The use of filler is one way of obtaining specific desirable properties in a rubber composite material, including those with electrical, thermal, mechanical or curing properties [16,17,18,19,20]. Regarding polymers other than rubber, the application of fillers in polymers is also effective. There are numerous reports of investigations applying filler to a polymer [21,22,23]. A method is thus needed for making a piezoelement of rubber utilizing a filler that is simple for engineering production, involving low cost and time, and having the durability needed to withstand impulsive, large or repeated forces, and large elasticity and extensibility. In addition, when the filler is magnetic, we can obtain additional effective properties for the intended engineering application. Therefore, in the present study, we propose a new piezoelement made of natural rubber that uses a magnetic compound fluid (MCF) [24] as magnetic filler and that is solidified under the application of electric and magnetic fields.

In order to accomplish the aim of this paper, we employ the MCF rubber technique [25]. Shimada devised MCF in 2001 [24] as a colloidal fluid containing spherical Fe_3_O_4_ particles on the order of 10 nm in size, as well as other metal particles such as Ni, Fe, Cu, etc., on the order of 1 μm. These particles are dispersed in a solvent such as water, kerosene, or silicone oil to compound a magnetic fluid (MF), which is an ordinary intelligent fluid responsive to the magnetic field due to the Fe_3_O_4_ particles. When a magnetic field is applied, the Fe_3_O_4_ particles play a bonding role among the metal particles, allowing numerous metal and Fe_3_O_4_ particles to aggregate [26]. MCF is useful for engineering applications owing to these magnetic clusters [27]. It is effective in polishing [28,29,30,31,32,33], as a material for use as a damper [34,35], and as a composite material, including MCF rubber. The composite material rubber mixed with MCF was called MCF rubber in previous studies. The magnetic clusters in MCF rubber produce the electric conductivity that leads to large changes in response to very small pressures.

MCF rubber material has been shown to be suitable for making silicon oil rubber [36,37,38,39,40] and natural rubber [41,42,43,44,45,46,47]. It can be solidified under a constant temperature (for convenience, we call this the “drying method”). In general, ordinary piezoelements made of rubber have needed the drying method when a curing agent such as sulfur is added for vulcanization. In the case of MCF rubber handled in the previous study [41,42,43,44,45,46,47] as well as in the present study, sulfur vulcanization has not been used. Even if just the drying method is used for curing, the rubber can become suitable for the application in sensing fields under very low force or pressure because the rubber has the potential to comprehensively meet the condition of linear elasticity at very low force or pressure. Naturally, an effect of the sulfur on the solidification of the MCF rubber can be expected under large force and pressure. In the present study, we shall make no further inquiry into the effect of sulfur on the properties of MCR rubber and may leave those details to another study. Because the human skin which corresponds to a robot skin is extremely sensitive under very low force or pressure, when the MCF rubber is solidified by the application of a magnetic field and the drying method, it has a switching effect, which means the electric resistance changes from high to low because of the magnetic clusters. Therefore, the MCF rubber has been demonstrated to be suitable for sensing concave and convex shapes when installed on a robot [45,46]. It has also been clarified that in the case of natural rubber, MCF rubber has more sensitivity than commercial pressure-sensitive electrically conductive rubbers. Natural rubber (NR-latex) is also superior for the previously mentioned sensor requirements of large elasticity and extensibility and MCF filling is also very effective for producing higher sensitivity piezoelements made of NR-latex. Therefore, in the present study, we adopted an NR-latex type MCF rubber. Furthermore we also employed reagent doping.

In general, the drying method for curing is inefficient for engineering production. To knead metal or carbon into the rubber requires considerable production costs and time because of the rolling machine and drying needed. In addition, the material can only be produced in sheets; other shapes are not possible due to the rolling. Therefore, Shimada devised a new method that did not involve the drying method [48,49,50]. It is such a convenient and simple method that low production costs and time can be achieved. It also allows for the creation of any shape. By the application of an electric field, the MCF rubber creates an anode plate as a thin film. Then, with the application of another a magnetic field, the thickness of the MCF rubber grows along the magnetic field line. Ordinarily, NR-latex without MCF is created as a thin film, and its thickness does not grow even after a long application of a electric field. Therefore, the creation of MCF magnetic clusters by the application of the magnetic field is needed. Since the magnetic field is applied along the same direction of the electric field line, the thickness of the MCF rubber grows. If only the electric field or magnetic field is used, the electrical properties are less sensitive than when both are used, and the resulting rubber is less extensible, as shown in previous studies [48,49,50]. Therefore, both the electric and magnetic fields are necessary for larger elasticity and extensibility. The new method is related to so-called electric polymerization which is positioned as one of the types of vulcanization. In summary, the curing of NR-latex is promoted by the alignment of magnetic clusters in the filler created by the application of a magnetic field under electric polymerization conditions.

On the other hand, in general, the effect of a piezoelement can be described as piezoresistivity or piezoelectricity. A piezoresistive element requires the application of voltage by a power supply and this produces the changes in resistivity of the piezoelement. This is essentially an electric conductor that allows electrons to percolate through the material. The mechanism of the electric conductivity is explained mainly by percolation theory [21] and tunnel theory. In the former theory the percolation threshold is a very significant factor. In the case of the large aggregation of particles or large aspect ratio of the particle shape, percolation theory cannot be applied because the percolation threshold decreases in spite of the enhancement of electric conductivity. Regarding the latter theory, when a voltage is applied to the material, the phenomenon can be explained by the proposition that electrons jump to percolate through the barrier of non-conductive material by a diode effect [40,51]. In MCF rubber, because the magnetic clusters are extremely aggregated, it has been proposed that the tunnel theory is suitable for the MCF rubber. On the other hand, piezoelectric elements does not require a power supply but rather obtain their charge from the changes in voltage induced from the material. This is a ferroelectric substance that allows the ions on crystal lattices sites with asymmetric charge surroundings within the material get closer to generate electricity.

Piezoelectricity is suitable for the piezoelements which are to be utilized in robotics because of the attainable weight reduction as mentioned above. The induced voltage indicates the approach of positively and negatively charged ions in the material with the application of force or pressure. Piezoelectricity is very significant for robot sensors.

However, we discovered that the MCF rubber dealt with in the present paper has both piezoelectric and piezoresistive effects. The relation between the former and the latter piezo effects has not yet been clarified. In robotics piezoresistive elements are also effective in some sensing applications. Therefore, it is valuable to investigate the MCF rubber property under the effects of both piezoelectric and piezoresistive effects.

From the noted above, in this paper, we first discuss the production of the MCF rubber piezoelements by our new method. We then clarify the properties of the response of the MCF rubber piezoelements in terms of piezoresistivity and piezoelectricity through measurement by pressing as meaning *d^33^* [52,53,54]. Next, we clarify the relation between the piezoelectric and piezoresistive effects. In addition, a hybrid piezoelement with the former and the latter piezoeffects is proposed. This hybridization means both piezoeffects have a role. The hybridization is useful for the sensing in robotics.

## 2. New Method of Producing a Piezoelement

The new method used in this study to produce MCF rubber applied to the manufacture of piezoelements has been detailed previously [48,49,50]. We therefore do not describe it here. MCF rubber liquid was poured onto one metal plate and sandwiched by another. Permanent magnets with a 15 mm × 10 mm rectangular surface were applied to the inside of the plates to form the outer surface of each plate. The applied magnetic field at the position of the MCF rubber liquid was 490 mT. An electric field held constant at 6 V and 2.7 A was supplied between the plates at 30-min periods. The plates were held apart at a constant thickness using a 3-mm spacer as an electrode gap. It is reasonable to reduce the quantity of the applied voltage, electric current and time, and to increase the spacer thickness. Therefore this new method led to the effective production of low cost and production time piezomaterial. The MCF rubber liquid was then vulcanized by application of the electric field.

We used plates made of stainless steel with a 35-mm square surface and 1-mm thickness, and conducted the experiment under atmospheric room conditions. We used three types of ingredients, as shown in Table 1. Here, Ni was a powder with μm-ordered twig-shaped particles (No. 123 by Yamaishi Co. Ltd., Noda, Japan), MF with 50 wt % of Fe_3_O_4_ (M-300, Sigma Hi-Chemical Co. Ltd., Tsutsujigasaki, Japan), and NR-latex (Rejitex Co. Ltd., Atsugi, Japan). Two types of reagent used as dopant were compounded into the MCF rubber for doping with KI or ZnO. The KI solution was an aqueous potassium iodide solution made up of 60 g of water and 40 g of potassium iodide. Doping was conducted before the application of the electric field.

**Figure 1:**
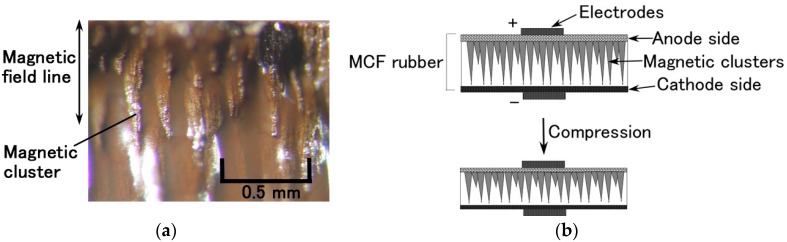
Cross section of Type 1 made of Type A: (**a**) photograph of magnetic clusters created in the vulcanized Type 1 MCF rubber by electric and magnetic fields; (**b**) schematic diagram of the compression of MCF rubber as a piezoelement.

**Figure 2:**
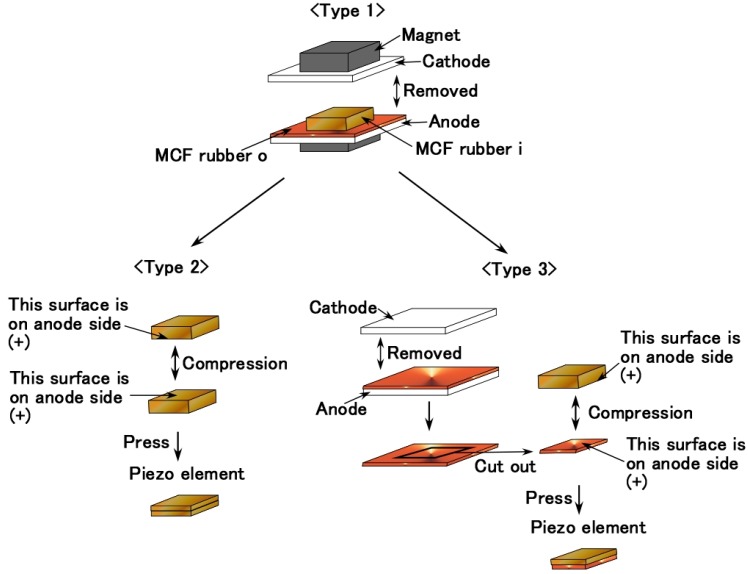
Production of layered piezoelements utilizing MCF rubber vulcanized by the application of electric and magnetic fields.

With this new method of producing MCF rubber for a piezoelement, we observed magnetic clusters aggregated by Ni and Fe_3_O_4_ particles like a needle (Figure 1a), confirming the results from a previous study in the case of Type 1 (as indicated on the top in Figure 2) made from Type A rubber [48,49,50]. The magnetic clusters occurred at the area where the permanent magnet was applied to the MCF rubber. Figure 1 shows a cross-section of the MCF rubber. The perpendicular direction is the applied magnetic field line, which was the same as the electric field. Therefore, we could assume compressed MCF rubber piezoelement was produced by the application of compression force, as shown in Figure 1b. By the compression, the positive and negative ions within the MCF rubber get reciprocally closer, inducing a voltage that can be measured by the electrodes placed on both outer surfaces, as shown in Figure 1b.

Figure 2 shows the principle of the production of a single piece of vulcanized MCF rubber as a piezoelement. Type 1 is a single-layer vulcanized MCF rubber. On the other hand, we can consider multiple-layer MCF rubber as other piezoelement. This is because we discovered an induced voltage between each area of the single vulcanized MCF rubber, shown below in Table 2. In other words, the MCF rubber is also a pyroelectric material. There have been few reported investigations of pyroelectric rubber comparing to polymers [55,56,57]. Therefore MCF rubber is a precious material. It made it possible to create a new kind of piezoelement by cutting out the optimal area from the MCF rubber and adhering them to each other.

**Figure 3:**
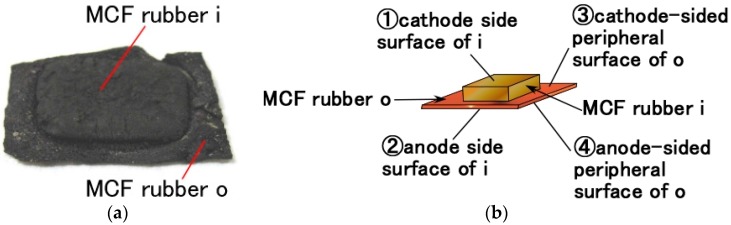
MCF rubber vulcanized by the application of electric and magnetic fields: (**a**) photograph of MCF rubber; (**b**) position of MCF rubber on the measurement of induced and electric resistance by connecting points.

Here, we therefore designate three types of piezoelement (Types 1–3), as follows: when a small quantity of MCF liquid was poured on the electrodes and electric and magnetic fields were applied, two areas of vulcanized MCF rubber were adhered on the electrode: thick MCF rubber on the magnetic field area or permanent magnet area, shown as “MCF rubber i” in Figure 2; and comparatively thin MCF rubber in the non-magnetic field area, i.e., outside the permanent magnet area, shown as “MCF rubber o” in Figure 2 (these are shown by a photograph in Figure 3a). MCF rubber i had the magnetic cluster configuration shown in Figure 1, while MCF rubber o did not. Therefore, we considered a combination of several MCF rubber i’s with magnetic clusters. At first, two MCF rubber i's were extracted from two MCR rubbers, as shown on the left in Figure 2. Next, these MCF rubber i's were combined, and the combined MCF rubber was called Type 2. Self-adhesion hardening was used without any adhesive to reduce superfluous costs. MCF rubber vulcanized by electric and magnetic fields has surfaces that can be combined face to face as shown in Figure 2, left. However, the different surfaces created by the anode and cathode electrodes cannot be adhered due to the difference of configuration between the surfaces of the MCF rubber on the anode and cathode, which is based on the different radical polymerization between the anode and cathode. On the other hand, MCF rubber vulcanized by electric and magnetic fields has two areas of MCF rubber, i and o. The induced voltage and electric resistance between the varied positions on these surfaces were measured, as shown in Figure 3. The results are listed in Table 2.

In the case of ①-②, by vulcanization with electric and magnetic fields, electric conductivity through the inner rubber was enhanced by electric polymerization, and the MCF rubber approached being an electric conductor, as reported in previous investigations [48,49,50]. In the case of ①-①, except for Type C, electric conductivity on the surface of MCF rubber i was no less high than that throughout the rubber in the case of ①-②. As for Type C, the electric conductivity in the case of ①-① was reduced owing to the ferroelectric substance ZnO. However, that in the case of ①-② with ZnO was enhanced, owing to the electric polymerization with dopant by the new method. Thus the results of ①-① differed by type of dopant, as shown in Table 2. In the cases of ①-② and ①-①, when the electric resistance was small, the induced voltage was very small. This was because MCF rubber becomes an electric conductor due to the magnetic clusters, the MCF filler, or the KI doping, and the ions disappear so that a voltage may not be induced by itself. In the cases of ①-③, ③-④ and ③-③, not only the type of percolation through the MCF rubber, but also the type of conduction over the surface of the MCF rubber created ferroelectric substances, because of the ions generated by the application of only the electric field. Therefore, the induced voltage exists and electric resistance becomes larger.

The results in Table 2 indicate the combination of MCF rubbers i and o, corresponding to ①-③ in Table 2, can be surmised effective. Therefore we can consider another combination of plural MCF rubbers. As shown on the lower right side of Figure 2, MCF rubber vulcanized by the electric field only, corresponding to MCF rubber o, was combined with MCF rubber i face to face on the surface vulcanized at the anode. This is called Type 3. The different surfaces on the anode and cathode electrodes cannot themselves be adhered, which is the same as Type 2. As for Types 2 and 3, two MCF rubbers were pressed together t 2.2 kg for 1 hour under 47 °C with a self-adhesion effect as shown in Figure 2. Table 3 shows the thickness of the created MCF rubber.

The measurement procedures for the analysis of the electrical and mechanical properties were described in previous studies [48,49,50]. In those studies, these properties were measured for robotics, the voltage or electric current inside the rubber at compression and the shear motions at the application of D.C. voltage were considered as the electrical properties, and the strain-stress response for the mechanical properties, during consecutive compressions. In the present study we measured the electrical property of the voltage or electric current on the MCF rubber at compression using the same NFE experimental apparatus as in the previous study [48,49,50]. We first measured the voltage between the electrodes without installing a power supply, and the electric resistance between electrodes, as shown in the designated figure in the previous study. We then measured the voltage between the electrodes when installed between the electrodes. The former measurement was for induced voltage related to piezoelectricity, and the latter for interior voltage related to piezoresistivity. The paired electrodes used had the same 3 mm × 3 mm square form.

## 3. Piezoelectric Response

**Figure 4:**
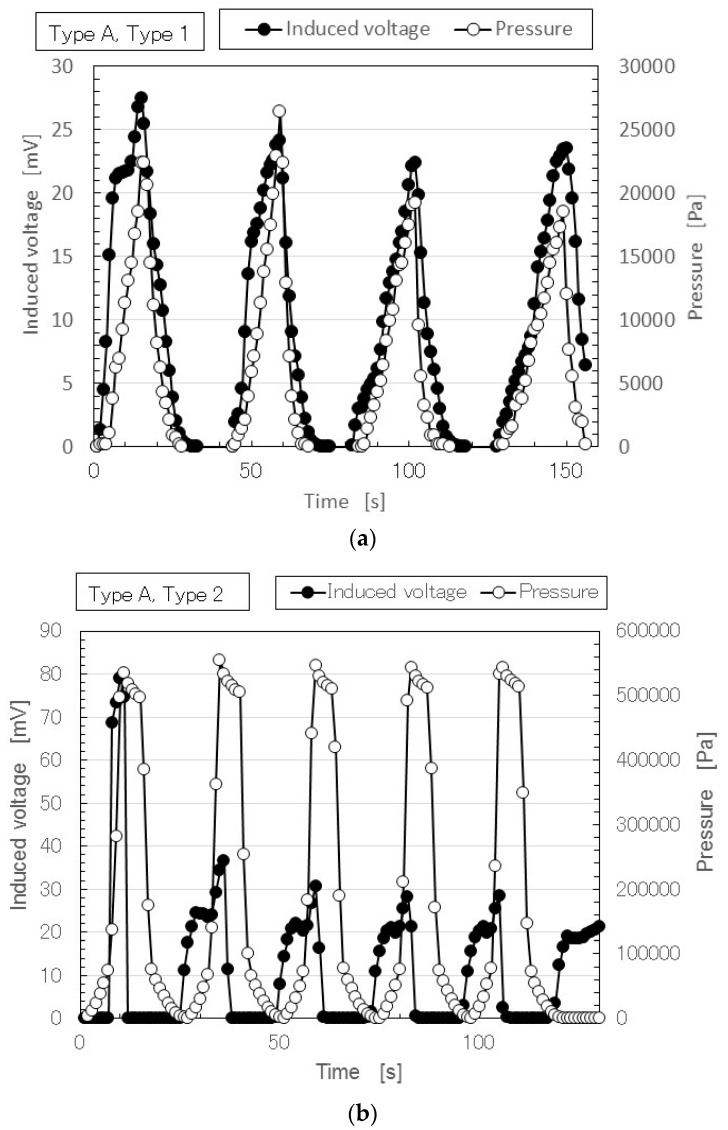

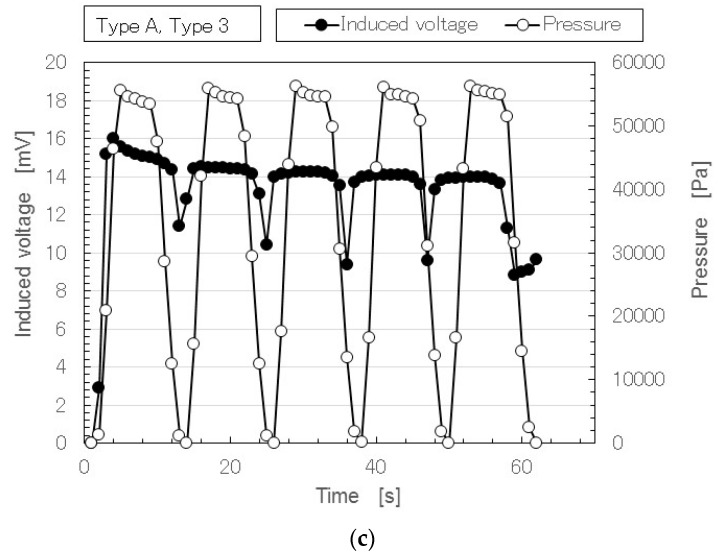
Typical results of time-lapse of induced voltage and pressure in the case of Type A. (**a**) Type 1; (**b**) Type 2; (**c**) Type 3.

At first, we investigated the experimental results without doping. Figure 4 shows a typical time variation result for the induced voltage with repeated application of pressure in the case of Type A.

**Figure 5:**
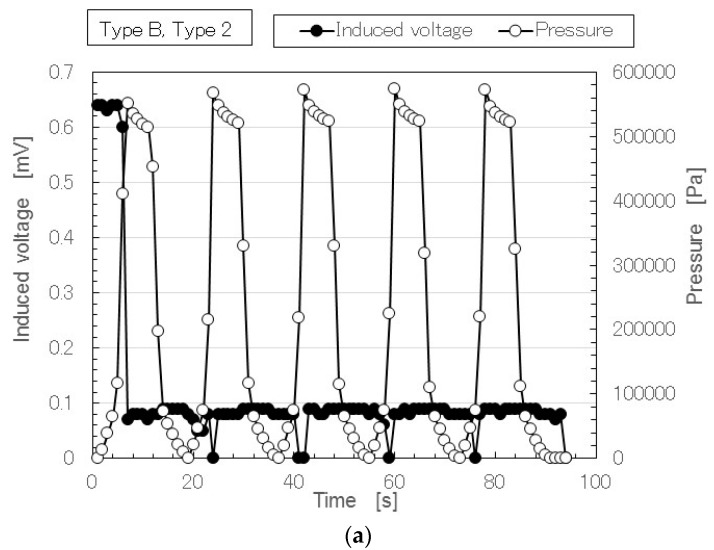

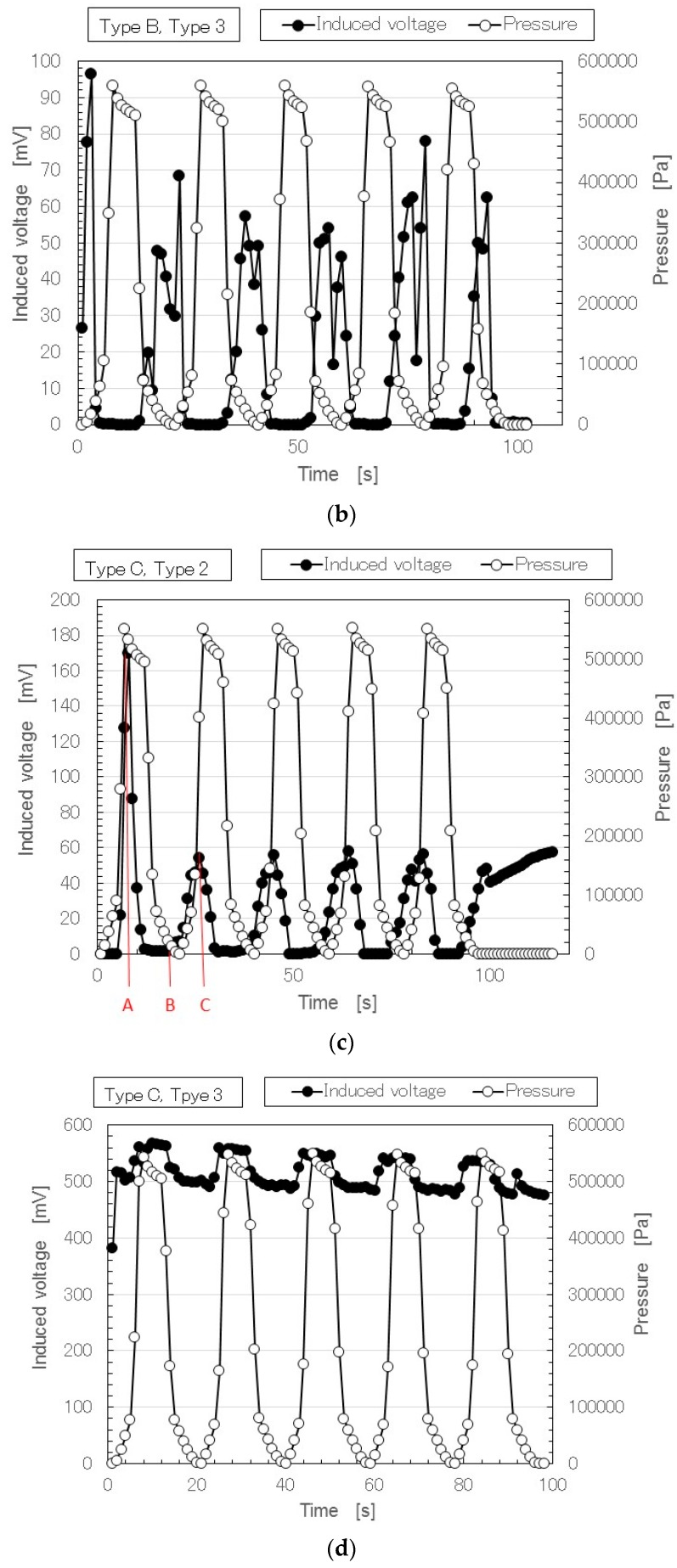
Typical results of time-lapse of the induced voltage and the pressure in the cases of Types 2, 3 and B, C. (**a**) Type B and 2; (**b**) Type B and 3; (**c**) Type C and 2; (**d**) Type C and 3.

The performance of the MCF rubber does not degrade over time with the repetition. The deviation of the measured induced voltage in the figure is within the allowable limit of error. Therefore the MCF rubber is stable. Furthermore it can be confirmed by the experiment that Types 2 and 3 has more stability than Type 1 and the cause is guessed to be due to the multilayer structure. Figure 4a is different from that of Types 1 and A in the previous study, which was conducted with an insulation paper sheet, 0.121-mm in depth and more than 100-MΩ in resistance, inserted between the MCF rubber and the upper electrode for stabilization of the induced voltage [50]. The voltage induced in the MCF rubber with doping had stable changes during the repetition, as shown in Figure 5, even if the MCF rubber did not have an insulation sheet. Therefore, in order to compare the MCF rubber with doping we show the case without an insulation sheet for Type 1. Regarding the sensitivity at small pressure, even with a different numbers of layers, the induced voltage had the same high sensitivity. The physical mechanism of the induced voltage at low pressure had the same qualitative tendency; its cause is shown below.

Next, we investigated the effect of doping on induced voltage. Figure 5 shows the results of MCF rubber Types B and C in Types 2 and 3. As denoted in Figure 4, in Figure 5 the performance of the MCF rubber also does not degrade over time with the repetition. The deviation of the measured induced voltage in the figure is within the allowable limit of error. Therefore the MCF rubber has stability. Doping enhanced the induced voltage. In particular, Type C was the largest. The induced voltage in Type 3 was larger than that in Type 2. This was due to the effect of the configuration of the piled layers.

As seen in Type of A and 3 in Figure 4c, in Type of B and 2 by Figure 5a and Type C and 3 in Figure 5d, the typical characteristics indicated that at small pressure the induced voltage was responsive to the pressure, while at large pressure it holds constant at a low value. This characteristic shows the same qualitative tendency regardless of the type of layer and type of dopant. The cause is due to the week vulcanization of the MCF rubber without involving sulphur. Therefore, the MCF rubber is compressed before it recovers to its initial size by the reduction of the pressure. If the sulphur is used, the vulcanization between the isoprene of the NR-latex is more solidified and the MCF rubber recovers to its initial size upon reduction of the pressure.

**Figure 6:**
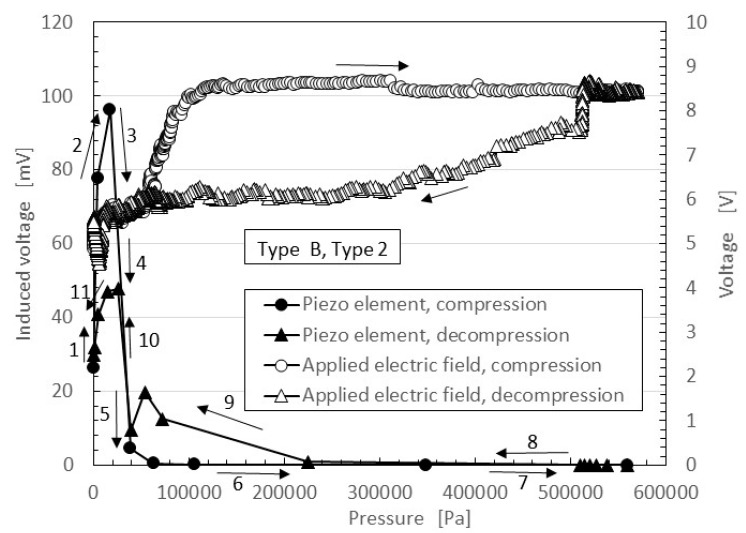
Typical results of the induced voltage and voltage flowing inside the MCF rubber by the application of a voltage.

**Figure 7:**
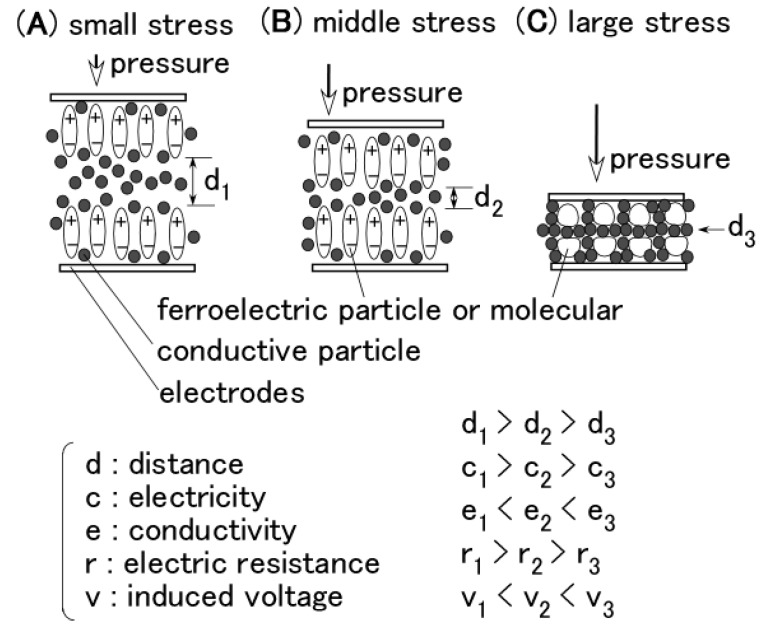
Schematic diagram of the physical model of Piezo effect of MCF rubber.

In Figure 4 and Figure 5, regarding the large responsivity of the induced voltage at small pressure and the small induced voltage at large pressure, the behavior of the ions inside the MCF rubber differed according to the amount of pressure. Figure 6 shows typical results of the comparison between the induced voltage (represented by black symbols) and the voltage flowing inside the MCF rubber with the application of a voltage (represented by white symbols). The former corresponded to piezoelectricity and the latter to piezoresistivity. The latter was measured by the application of a DC 10 V power supply using the same NFE experimental apparatus used in the previous study [48,49,50]. The arrows with numbers in the figure indicate the order from compression to decompression. The result is one cycle of repetition. Because the MCF rubber has a high sensing response at very small pressure, the induced voltage occurs around 0 Pa and is enhanced up to around 20 kPa by the compression, as indicated from 1 to 2 in the figure. Corresponding to this is the situation from 0 s to A, marked in red in Figure 5a. The qualitative tendency of Figure 5c, as is shown in the previous discussion of Figure 4 and Figure 5, and is the same as that in the other figures, including Figure 5, and directly designated in Figure 6. The cause is the fact the positive and negative ions inside the MCF rubber approach but do not contact, as shown in Figure 7a.

Figure 7 shows the physical mechanism of the induced voltage. The illustration shows the MCF rubber is a ferroelectric substance with smaller electricity, or where the electric resistance is larger with smaller conductivity. At more than 20 kPa in Figure 6, the induced voltage is decreased as indicated from 3 to 7, because the positive and negative ions come into contact, and the Ni particles aggregate such that the electron can pass through the MCF rubber, as shown in Figure 7c. Corresponding to this is the situation from A to B, indicated in red in Figure 5c. The MCF rubber is a conductor with smaller electric resistance and with smaller electricity. Therefore, the voltage flowing within the MCF rubber upon application of a voltage is larger than that at less than 20 kPa. By decompression, the MCF rubber then transits from being a conductor to being a ferroelectric substance, as indicated from 8 to 11 in Figure 6, or from B to C, indicated in red in Figure 5c. As a result, in MCF rubber, piezoelectricity occurs under small pressures and piezoresistivity at high pressure. The MCF rubber is thus a hybrid of a ferroelectric substance and a conductor.

Because the aim of the present report is the use of rubber with filler, we compared these results to those of piezoelements just made of rubber with filler as follows: in the case of MCF rubber from Figure 6, as for piezoelectricity the effective piezoelectric coefficient *d^33^* is estimated to be on the order of 10^−10^ C/N which was measured by referring the study [54], and as for piezoresistivity, the electric conductivity ranged from 10^−4^ to 10^−1^ S/m.

As for piezoelectricity, the effective piezoelectric coefficient *d^33^* in the case of chloroprene rubber consisting of PbTiO_3_ ceramic powder with 127-mm square plate is 10^−11^ C/N order [12]. *d^33^* in the case of silicon oil rubber using together zirconate titanate (PZT) particles with cylinder shapes having 50-mm diameter and 2-mm thickness is on the order of 10^−11^ C/N [16]. *d^33^* in the case of silicon oil rubber combined by PZT particles of cylinder shape having 50-mm diameter and 2-mm thickness is 10^−12^ C/N [18].

As for piezo-resistivity, the electric conductivity of acrylonitrile butadiene rubber containing furnace carbon black is from 10^−8^ to 10^−2^ S/m, and the order changed with the carbon black volume fraction [14]. The electric conductivity of NR-latex combined by sodium monmorillonite and polypyrrole is from 10^−4^ to 10^−2^ S/m, and also changed with the loading of the nanocomposite [15].

From the comparison with the above results, the piezoelectric and piezoresistive effects in the MCF rubber piezoelements are larger than those of other filled rubber piezoelements.

## 4. Verification of the Piezoeffect of MCF Rubber

In the last section, we verify the suitability of the MCF rubber created here in contrast to the conventional piezoelements with filler and dopant, which are the traditional methods of enhancing the piezoeffect. However, those materials have always been produced by the drying method as previously described. In contrast to the conventional piezoelements, the greatest feature of the present MCF rubber production method is the use of both magnetic and electric fields for vulcanization. In order to compare with conventional piezoelements, the creation of MCF rubber without the aid of the electric field should be considered. In order to verify the superiority of the MCF rubber produced in the present study, we created other MCF Type 1 rubbers with filler and dopant using only the drying method, without the application of an electric field for vulcanization. Ni and Fe_3_O_4_ were used as fillers, corresponding to Type A. The doping was the same as that used in Types B and C. We also dealt with two types of MCF rubber, both with and without the application of a magnetic field during vulcanization. The drying time requires 24 h for the former MCF rubber and 48 h for the latter. These differences were due to the effect of the alignment of Ni and Fe_3_O_4_ particles created by the magnetic field to promote vulcanization.

**Figure 8:**
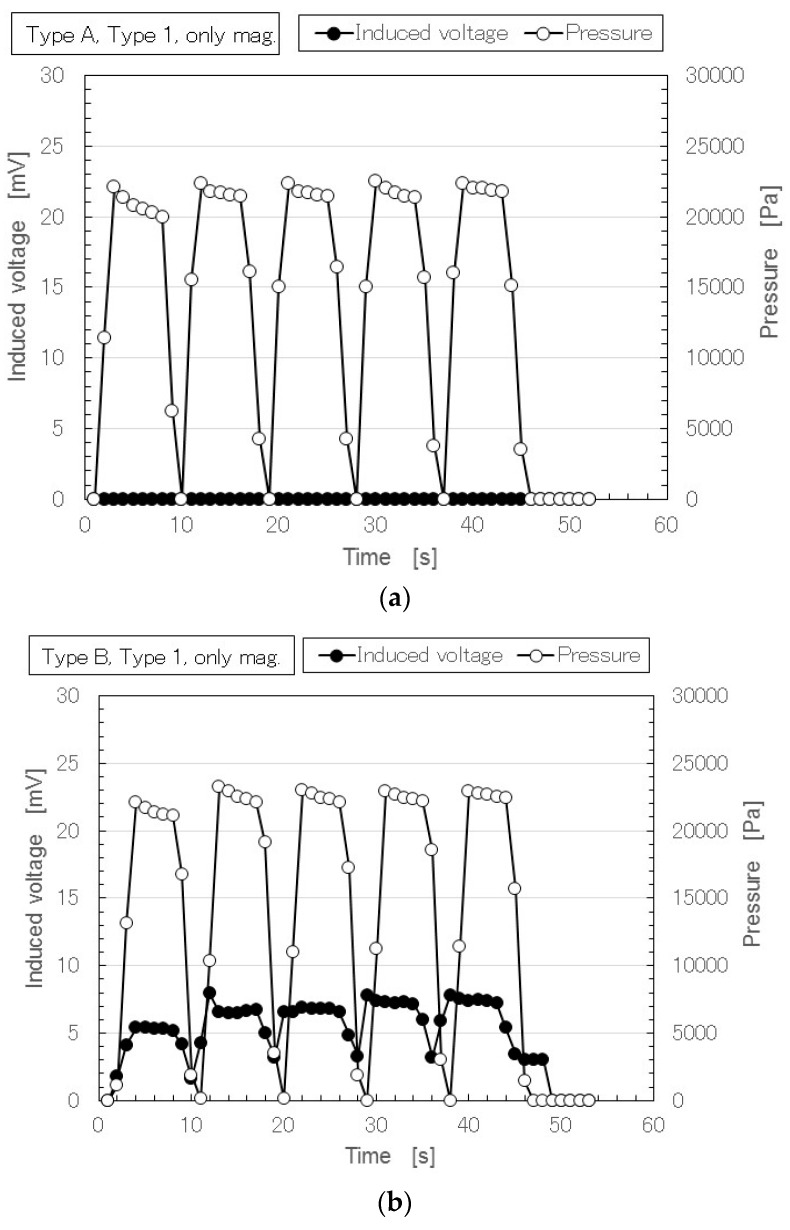

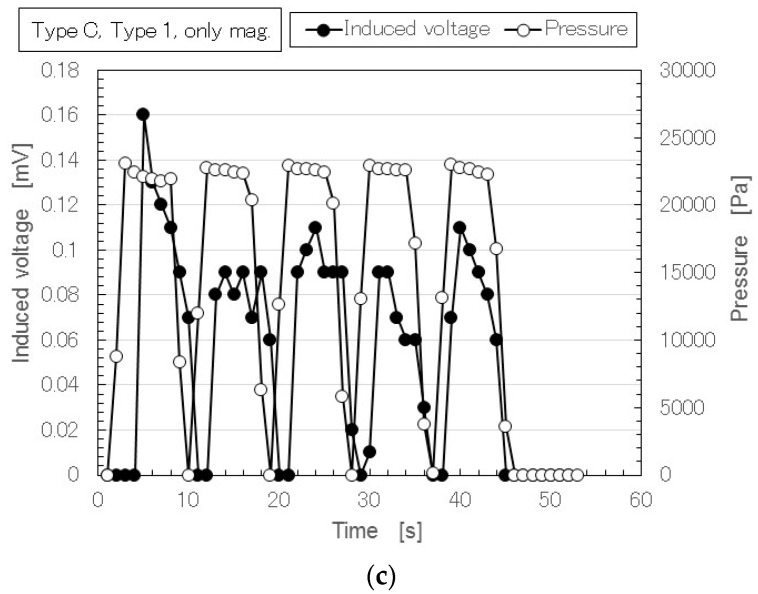
The results of time-lapse of the induced voltage and the pressure on MCF rubber created with the drying method only. (**a**) Type A; (**b**) Type B; (**c**) Type C.

Figure 8 shows the time variation of the induced voltage under repeated application of pressure in the case of the MCF rubber created without using an electric field. On the other hand, as for Types A, B and C created without either a magnetic field or electric field, the induced voltage of each Type was zero (results not shown).

Types A and 1 are the MCF rubbers corresponding to the conventional piezoelements with filler and without dopant, and without or with a magnetic field. The induced voltage with a magnetic field as shown in Figure 8a was zero. Therefore, the voltage was not induced by the filler that forms as magnetic clusters. In other words, magnetic clusters alone did not generate induced voltage without doping, or, voltage was not induced by filler that did not form magnetic clusters. Next, we prepared a conventional piezoelement with filler and dopant, corresponding to Types B and C without an electric field, and without or with a magnetic field. Figure 8b shows that Type B MCF rubbers with a magnetic field had a large induced voltage. On the other hand, Figure 8c shows that the Type C MCF rubbers with a magnetic field had no more than induced voltage. These findings were due to the effects of the doping on the configuration of the magnetic clusters. However, in the case of doping without a magnetic field, the voltage was not induced by the filler alone that did not form magnetic clusters, as previously described. Therefore, the effect of doping on the magnetic clusters generated the induced voltage.

In the case without doping, Figure 4a shows the MCF rubber in Types A and 1 that involve MCF rubber i (as shown in Figure 2) consisting of magnetic clusters. In this case, an induced voltage was generated. By comparing this result to Figure 8a, we can hypothesize that the result was due to electric polymerization with the aid of the electric field for vulcanization. Therefore, even if we do not use dopant, we can obtain a large induced voltage by electric polymerization. The electric polymerization has a role in generating an induced voltage in the MCF rubber.

Meanwhile, the results of Figure 4, Figure 5 and Figure 8 suggest another conclusion regarding Types 2 and 3. The piezoelements of MCF rubbers in Types 2 and 3 (see Figure 5) involve Type 1, which corresponds to MCF rubber i shown in Figure 2, consisting of magnetic clusters and dopant. Therefore, it is possible to clarify the effect of the magnetic clusters with dopant on the piezoelectric response by comparing the MCF rubbers with and without a magnetic field in the case of dopant. The voltage generated in the case of the MCF rubber created with the help of an electric field (Figure 5) is the same as that of the MCF rubber created with a magnetic field and without an electric field (Figure 8b,c). In Figure 8b,c, the effect of dopant on the magnetic clusters generated the induced voltage, as already mentioned. Therefore, in the case of the MCF rubber as in Figure 8b,c, we can determine the effect of dopant on the magnetic clusters generated the induced voltage. The MCF rubbers in Types 2 and 3 were due to the effects of the magnetic clusters and the doping. Thus, the generating factor of piezoelectricity in the MCF rubber has synergetic effects with the magnetic clusters, doping, and electric polymerization.

## 5. Conclusions

The results from the consecutive experimental procedures can be summarized as follows. First the proposed MCF rubber piezoelements with two types of multilayered structure have two typical characteristics as follows:

1. As shown the typical results of Figure 4c and 5a,d, the typical characteristics of the piezoelement made of natural rubber with filler as MCF created with the help of electric and magnetic fields indicates that at small pressure, the induced voltage is responsive to the pressure, and at large pressure it holds constant at a low value. The cause is due to the weak vulcanization of the MCF rubber without involving sulphur. Therefore, if we desire responsivity at a small pressure, we only have to produce the MCF rubber without sulphur. This characteristic has the same qualitative tendency as the type of layer and kind of dopant.
2. As shown the typical results of Figure 5b,c or Figure 6, at small pressure the induced voltage is highly responsive, but the conductivity is small; in other words, the MCF is a ferroelectric substance. The MCF rubber presents piezoelectricity. On the other hand, at large pressure the induced voltage is smaller, but the electric resistance is decreased such that the MCF rubber becomes a conductor. The MCF rubber presents piezoresistivity. Thus, the piezoeffect varies depending on the amount of pressure. This typical characteristic has the same qualitative tendency on the type of piled layers and the kind of dopant.

Concerning these characteristics as shown above in 2, the MCF rubber is a piezoelement hybrid with piezoelectricity and piezoresistivity. The hybrid piezoeffect is suitable for application of various engineering fields. These piezoelectric and piezoresistive effects can be utilized by changing the quantity of the pressure in robotics by some method.

In addition, multilayered MCF rubber of Types 2 and 3 has more stability than the single-layered one designated as Type 1. The piezoelectricity estimated by the effective piezoelectric coefficient and the piezoresistivity by the electric conductivity of the MCF rubber piezoelement are superior to those of other filled rubber piezoelements.

Secondly, regarding the piezoelectricity of the MCF rubber, we clarified how the induced voltage is generated by the magnetic clusters, doping, and electric polymerization as follows: as denoted the typical results of Figure 8a, the effect of magnetic clusters alone does not generate an induced voltage without doping. However, as denoted the comparison of typical results of 4a and Figure 8a, electric polymerization has a role in generating the induced voltage of MCF rubber. On the other hand, as denoted the typical results of Figure 8c, the effect of dopant on the magnetic clusters generates an induced voltage. This tendency does not depend on the type of piled layers. Thus the generating factor of piezoelectricity in MCF rubber shows synergetic effects between the magnetic clusters, doping, and electric polymerization.

## Figures and Tables

**Table 1:** Ingredients of the MCF rubber liquid used in this study.

Type A	Type B	Type C
Ni: 6 g	Ni: 9 g	Ni: 3 g
MF: 1.5 g	MF: 2.25 g	MF: 2.25 g
NR-latex: 6 g	NR-latex: 9 g	NR-latex: 9 g
-	KI solution: 6.75 g	ZnO: 3 g

**Table 2:** The electric resistance and induced voltage by connecting different points of MCF rubber: Ω designates electric resistance and mV the induced voltage.

	①-②		①-①		①-③		③-④		③-③	
	Ω	mV	Ω	mV	Ω	mV	Ω	mV	Ω	mV
Type A	0.9	0	8.7	0	1.8 M	95	890 k	36	11.8 M	30
Type B	2.1	0	26	0.1	340 k	220	700 k	200	500 k	230
Type C	5.4	0	30 k	120	2.0 M	75	3.0 M	60	2.8 M	37

**Table 3:** Thickness of created MCF rubber.

Rubber	Type 1 (mm)	Type 2 (mm)	Type 3 (mm)
Type A	3.00	3.00	1.90
Type B	3.00	2.50	2.50
Type C	3.00	3.00	3.00
